# Child maltreatment exposure and adolescent nonsuicidal self-injury: the mediating roles of difficulty in emotion regulation and depressive symptoms

**DOI:** 10.1186/s13034-023-00557-3

**Published:** 2023-01-28

**Authors:** Changchun Hu, Jialing Huang, Yushan Shang, Tingting Huang, Wenhao Jiang, Yonggui Yuan

**Affiliations:** 1 School of Medicine, Southeast University, Nanjing, China; 2 Department of Psychosomatics and Psychiatry, Zhongda Hospital, School of Medicine, Southeast University, No. 3, Xinmofan Road, Nanjing, 210009 China; 3 Department of Clinical Psychology, Affiliated Hangzhou First People’s Hospital, Zhejiang University School of Medicine, Hangzhou, China; 4 Jiangsu Provincial Key Laboratory of Critical Care Medicine, Zhongda Hospital, School of Medicine, Southeast University, Nanjing, China

**Keywords:** Nonsuicidal self-injury, Adolescents, Child maltreatment, Difficulty in emotion regulation, Depressive symptoms

## Abstract

**Background:**

Although child maltreatment (CM) experiences are recognized risk factors for nonsuicidal self-injury (NSSI), the mechanisms underlying this relationship remain unclear. The purpose of this study was to examine whether difficulty in emotion regulation (DER) and depressive symptoms mediate the relationship between child maltreatment experiences and NSSI severity, adjusting for demographic variables.

**Methods:**

The participants were 224 adolescent inpatients recruited from a hospital in China (mean age 15.30 years, *SD* = 1.83; 78.6% females). Study measures included the Clinician-Rated Severity of Nonsuicidal Self-Injury (CRSNSSI), Childhood Trauma Questionnaire (CTQ-SF), Difficulties in Emotion Regulation Scale (DERS), and Patient Health Questionnaire-9 (PHQ-9). The hypothesized chain mediation model was tested using the structural equation model.

**Results:**

A total of 146 (65.18%) adolescents reported engaging in NSSI during the past 12 months, and 103 (45.98%) participants met the DSM-5 diagnostic criteria for NSSI. Emotional neglect (48.1%) and emotional abuse (46.1%) had the highest prevalence, followed by physical neglect (43.1%) and physical abuse (24.1%), whereas sexual abuse (12.5%) was the least prevalent form of CM. Separately, both DER and depressive symptoms significantly mediated the association between CM and NSSI, with DER being the strongest mediator, with an indirect effect of 49.40% (*p* = 0.014). At the same time, we also proved a potential chain-mediated pathway of DER and depression in the relationship between CM and NSSI.

**Conclusion:**

Child maltreatment seems to play a role in the aetiology of NSSI. DER and depressive symptoms both have a mediating role in the relationship between CM and NSSI. Importantly, DER seems to be a mediator with a stronger indirect effect compared to depressive symptoms.

**Supplementary Information:**

The online version contains supplementary material available at 10.1186/s13034-023-00557-3.

## Introduction

Nonsuicidal self-injury (NSSI) refers to deliberately damaging one’s body tissue without lethal intent; NSSI typically involves cutting, scratching, burning, and banging [1]. Epidemiological research consistently indicates higher prevalence rates of NSSI in teenagers than in adults [2]. The overall lifetime prevalence of NSSI is 19.4–26.7% among adolescents, and it is more common in girls than in boys (risk ratio 1.72) [3, 4]. NSSI is increasingly recognized as a significant public health concern because of its high prevalence and its association with several internalizing and externalizing disorders [5] and NSSI is considered to be a strong predictor of suicidal behaviour [6]. Identifying risk factors for adolescent NSSI is critical to understanding its mechanism and providing early prevention and treatments.

Growing research focuses on the potential mechanisms of child maltreatment (CM) experiences leading to NSSI. As distal risk factors for NSSI [1], CM includes five types: emotional abuse (EA), physical abuse (PA), sexual abuse (SA), emotional neglect (EN), and physical neglect (PN). A recent meta-analysis showed that CM and its subtypes are associated with NSSI, with the exception of childhood emotional neglect [7]. CM is generally considered a risk factor for many psychopathological and behavioural problems. A substantial amount of research has demonstrated that CM experiences are associated not only with higher rates of NSSI [8–10] but also with depression [11] and difficulty in emotion regulation (DER) [12]. Moreover, recent data also suggest that these two variables have mediating properties in the relationship between CM and NSSI. Brown et al. [9] found a partial mediating effect of EA and full mediating effects of SA and PN by depression on NSSI. Similarly, Titelius et al. [13] found that DER mediated the relationship between CM and NSSI frequency in a small clinical sample. Finally, DER partially mediated the effects of an invalidating family environment (a latent factor consisting of CM) and NSSI [14]. Thus, depressive symptoms and DER may indeed be important factors explaining the pathway from CM to self-injury. However, these proposed mediators often cooccur, and fewer studies have tested mediating effects when all variables are assessed simultaneously. Shenk et al. [15] found that when all variables (posttraumatic stress symptoms, depressive symptoms, and psychological dysregulation) were included in the same model, only posttraumatic stress symptoms mediated the relationship between CM and self-injury. Moreover, moderating factors such as sex and how CM and NSSI is measured also play a role between CM and NSSI [16]. Therefore, the multifactorial mediating mechanism between CM and self-injury needs further research to complement the current literature.

Multiple theoretical models of NSSI have been proposed to explain self-injury. However, numerous theories and related studies have converged upon defining NSSI as a problem of emotion dysregulation. Research focusing on the function of NSSI suggests that adolescents may engage in NSSI to reduce negative affect [17] and avoid unwanted emotions [18]. These findings are consistent with Linehan’s Biosocial Model of emotion dysregulation [19] postulates that invalidating environments contribute to deficits in emotion-regulating capacities, which increase the likelihood of engaging in NSSI behaviour to cope with distress. DER arises from biological anomalies combined with exposure to dysfunctional environments throughout development [20]. This theoretical model corresponds to a broader literature in which CM is frequently identified as an invalidating environment associated with various poor outcomes, including DER, depression, and NSSI [8, 10, 11]. To extend Linehan’s Biosocial Model above, Gratz and Roemer propose the “difficulties in emotion regulation” (DER) model [21], which addresses several deficiencies in emotion regulation (e.g., impulse control difficulties, self-perceived limited access to strategies, a lack of emotional clarity). Recent research has found that these aspects of DER are mechanisms of change in DER-model-based treatment for adolescents with NSSI disorder [22]. Despite robust relations among CM, DER, and NSSI, additional research is warranted to delineate the specificity of how DER might mediate relations from CM to NSSI.

The current study examined relations between CM and NSSI within a diverse sample of boys and girls in a psychiatric hospital setting. Considering the diagnosis of patients was mainly depressive disorders in our sample, additional research is warranted to delineate the specificity of how depressive symptoms might mediate relations from CM to NSSI. First, we tested the mediating role of DER and depressive symptoms in these relations. Second, we further tested the mediating chain effect of DER and depression from CM to NSSI. The previous research emphasizes that DER precedes the onset of depressive symptoms and can predict depressive symptom trajectory from early to middle adolescence [23]. Thus, the DER might mediate the relationship between childhood emotional abuse and current depression [24]. See Additional file 1: Figure S1 for details of the theoretical model. Simultaneously, each direct or indirect path was estimated to determine its effect size in explaining the pathway from CM to NSSI.

The present study investigated adolescent NSSI within a significant clinical sample through robust structural equation modeling [25]. In particular, to focus on NSSI with clinical significance and accurately describe its severity, the severity of NSSI behaviors by clinicians use the Clinician-Rated Severity of NonSuicidal Self-Injury (CRSNSSI-DSM-5) through structured interviews. To our knowledge, this is the first study identifying the mediating pathways of depressive symptoms and DER from childhood maltreatment to NSSI through a chain mediation model in adolescents, further elucidating the roles of DER for NSSI. According to the mentioned literature, DER is a mediator with a stronger effect. The current study represents an essential empirical contribution to the biosocial theory of NSSI.

## Materials and methods

### Participants and procedure

A total of 245 adolescents from the Affiliated Hangzhou First People’s Hospital of Zhejiang University in Hangzhou, China were enrolled in our study. The participants were adolescents (aged 12 to 18 years) admitted to a psychiatric inpatient unit. The recruitment period lasted more than 2 years, from August 2019 to November 2021. Two trained clinical psychiatrists independently conducted interviews to collect patients’ psychiatric histories and made diagnoses for the same patients according to the Diagnostic and Statistical Manual of Mental Disorders, fifth edition (DSM-5) criteria. The exclusion criteria were a current or past diagnosis of schizophrenia spectrum disorders, mental retardation, or gross cognitive impairment. Eligible participants and their guardians were asked whether they were willing to participate. After providing their written informed consent, the participants were asked to complete a comprehensive questionnaire to collect demographic information and data on child maltreatment, emotion regulation difficulties, and the severity of depression. The severity of NSSI behaviours was assessed by clinicians using the Clinician-Rated Severity of NonSuicidal Self-Injury (CRSNSSI-DSM-5) through structured interviews at hospital admission. The study was conducted in accordance with the Declaration of Helsinki and approved by the ethics committee of the Affiliated Hangzhou First People’s Hospital of Zhejiang University (IRB: 2020-K008-01, January 2020).

Twenty-one participants were excluded because they did not complete the questionnaire. Ultimately, 224 participants were included in the current analysis. In this study, the mean age of the sample was 15.30 years (*SD* = 1.83, range: 12–18 years), 78.6% were female, and the mean education was 9.54 years (*SD* = 1.97, range: 6–13 years). Approximately 31.2% of the sample had more than one diagnosis, including depressive disorders (72.3%), anxiety disorders (18.8%), and trauma and stressor-related disorders (18.8) (Table 1).

**Table 1:** Demographic and psychological characteristics of the study participants (n = 224)

Variable		*Mean (SD)/n (%)* (Valid) Value
Age (years), *Mean (SD)*		15.30 (1.83)
Sex	Boy, n *(%)*	48 (21.4%)
	Girl, n *(%)*	176 (78.6%)
Education (years), *Mean (SD)*		9.54 (1.97)
DERS, *Mean (SD)*		119.30 (22.69)
Childhood maltreatment	EA, *Mean (SD)*	12.80 (5.21)
	PA, *Mean (SD)*	7.87 (3.63)
	SA, *Mean (SD)*	6.11 (2.69)
	EN, *Mean (SD)*	14.60 (5.49)
	PN, *Mean (SD)*	9.62 (4.18)
Depression	PHQ-9, *Mean (SD)*	18.33 (5.45)
NSSI (CRSNSSI)	None, n *(%)*	78 (34.8%)
	Subthreshold, n *(%)*	43 (19.2%)
	Mild, n *(%)*	17 (7.6%)
	Moderate, n *(%)*	40 (17.9%)
	Severe, n *(%)*	46 (20.5%)
Diagnosis	Depressive disorders, n *(%)*	162 (72.3%)
	Anxiety disorders, n *(%)*	42 (18.8%)
	Trauma and stressor-related disorders, n *(%)*	42 (18.8%)
	Bipolar and related disorders, n *(%)*	23 (10.3%)
	Borderline personality disorder, n *(%)*	14 (6.3%)
	Disruptive, Impulse control and conduct disorders, n *(%)*	12 (5.4%)
	Obsessive compulsive disorders, n *(%)*	5 (2.2%)

*DERS* Difficulties in Emotion Regulation Scale, *PA* physical abuse, *PN* physical neglect, *EA* emotional abuse, *EN* emotional neglect, *SA* sexual abuse, *CRSNSSI* Clinician-Rated Severity of NonSuicidal Self-Injury

### Measures

#### Clinician-rated severity of nonsuicidal self-injury (CRSNSSI)

The Clinician-Rated Severity of Nonsuicidal Self-Injury measure was developed by the American Psychiatric Association and assesses the severity of nonsuicidal self-injurious behaviours or problems experienced by an individual in the past year. Clinicians complete the measure using a 5-point scale (Level 0 = None; 1 = Subthreshold; 2 = Mild; 3 = Moderate; and 4 = Severe). Each level is described as follows: None (No NSSI acts or NSSI acts on fewer than 3 days and no urge to self-injure again); Subthreshold (NSSI acts on 2–4 days or has self-injured in the past for 5 or more days and has reported urges to self-injure again); Mild (NSSI acts on 5–7 days using a single method and has not required surgical treatment); Moderate (NSSI acts on 8–11 days using a single method and has not required surgical treatment [other than cosmetic] or NSSI acts on 5–7 days using more than one method); and Severe (At least 1 NSSI act that required surgical treatment [other than cosmetic], NSSI acts on 12 or more days using a single method or NSSI acts on 8 or more days using more than one method) [26].

#### Childhood trauma questionnaire-short form (CTQ-SF)

The CTQ-SF is administered to assess CM in participants [27]. It includes the following five dimensions: physical abuse (PA), physical neglect (PN), emotional abuse (EA), emotional neglect (EN), and sexual abuse (SA). Respondents are asked to rate each item on a 5-point Likert-type scale from 1 (never true) to 5 (very often true). High scores indicate high levels of childhood maltreatment. The Chinese version of this questionnaire has been demonstrated to be a reliable and valid measurement tool [28]. In order to reflect the distal risk characteristic of CM, and try to reduce the impact of recent confounding factors, it accounts for maltreatment before the age of 12 years. We determined cut-off scores for the CM subscales based on previous studies, patients are identified as positive for CM if any one of these subscales exceeds the cut-off score: EA > 12, PA > 9, SA > 7, EN > 14, and PN > 9 [29]. Each subscale demonstrates good to excellent internal consistency (Cronbach’s alpha = 0.723–0.933).

#### Difficulties with emotion regulation scale (DERS)

The DERS [21], which includes 36 self-report items, is used to assess the following six emotion regulation difficulties: nonacceptance of emotions, difficulties engaging in goal-directed behaviour, impulse control difficulties, a lack of emotional awareness, self-perceived limited access to strategies, and a lack of emotional clarity. All 36 items are scored using a five-point scale from 1 (Rarely) to 5 (Almost always). The Chinese version of this questionnaire has been demonstrated to be a reliable and valid measurement tool [30]. The DERS total score is used as an indicator of emotional regulation difficulties. Cronbach’s alpha for the DERS in the present study was 0.957.

#### Patient health questionnaire-9 (PHQ-9)

The PHQ-9 is a self-administered instrument that was developed by Kroenke, Spitzer, and Williams [31] to measure the severity of an individual’s depression by evaluating depressive episodes using nine criteria based on the Diagnostic and Statistical Manual of Mental Disorders, Fourth Edition (DSM-IV). The scale consists of nine items that assess the frequency with which nine depressive symptoms have occurred in the past 2 weeks, with items rated on a 4-point Likert scale from 0 (not at all) to 3 (nearly every day). The scale is recommended by the Guidelines for Adolescent Depression in Primary Care (GLAD-PC) as an assessment tool for depression in adolescents [32]. The Chinese version of the PHQ-9 has demonstrated good psychometric properties in adolescent samples [33].

### Data analysis

We first used SPSS 26.0 to compute descriptive statistics for all study variables and the bivariate correlations between them. Next, we tested the hypothesized chain mediation model with structural equation modelling using Amos 26.0. No significant correlation was found between NSSI and SA. Therefore, SA was removed from the mediation analysis. In this model, CM (as indicated by EA, PA, EN, and PN) served as a predictor variable; the observed variables of DER (as indicated by the DERS total score) and the severity of depression (as indicated by the PHQ-9 score) served as mediating variables; and the observed variable of the severity of NSSI behaviours served as the outcome variable. Model fit was assessed using multiple complementary fit indices. These indices included the comparative fit index (CFI), Tucker Lewis index (TLI), and root mean square error of approximation (RMSEA). The cut-off criteria for a well-specified model are a CFI > 0.95, a TLI > 0.95, and an RMSEA < 0.06, and the cut-off criteria for an acceptable model are a CFI > 0.90, a TLI > 0.90, and an RMSEA < 0.08 [34]. Mediation was considered significant if the 95% CI of the indirect effect did not include zero. Bootstrapped confidence intervals (CIs) based on 5000 bootstrapped samples were used to determine the significance of indirect effects.

## Results

### Descriptive statistics

A total of 224 adolescents (48 boys and 176 girls) were included in the current analysis, with a mean age of 15.30 years (*SD* = 1.83). A total of 146 (65.18%) adolescents reported engaging in NSSI during the past 12 months, 88 (39.29%) adolescents reported engaging in NSSI during the past month, and 103 (45.98%) adolescents met the DSM-5 diagnostic criteria for NSSI. The prevalence of CM in our sample was 70.80%. EN (48.1%) and EA (46.1%) had the highest prevalence, followed by PN (43.1%) and PA (24.1%), whereas SA (12.5%) was the least prevalent form of CM (Table 1).

### Correlation analyses

Correlational analyses were conducted to examine the relationships among the variables of interest (see Table 2). All CM types (EA, PA, SA, EN, and PN) were significantly positively associated, and the correlation coefficients ranged from 0.185 to 0.739 (*P* < 0.01). The correlation between CM (EA, PA, EN, and PN, but not SA) severity (independent variable) and NSSI severity (outcome variable) ranged from 0.37 to 0.55 (*P* < 0.01). The correlation coefficients between the postulated mediators (DER and depression) and CM ranged from 0.149 to 0.662 (*P* < 0.01). The correlations between NSSI severity and the mediators (DER, depression) were 0.633 and 0.698 respectively. Among the covariates, only sex was associated with NSSI severity (*r* = 0.283, *P* < 0.01), age was not.

**Table 2:** Bivariate correlations between study variables

	NSSI	DER	EA	PA	SA	EN	PN	Depression	Sex
DER	0.633^b^								
EA	0.552^b^	0.656^b^							
PA	0.371^b^	0.399^b^	0.604^b^						
SA	0.083	0.178^b^	0.296^b^	0.312^b^					
EN	0.494^b^	0.585^b^	0.665^b^	0.420^b^	0.185^b^				
PN	0.391^b^	0.444 ^b^	0.658^b^	0.500^b^	0.243^b^	0.739^b^			
Depression	0.698^b^	0.793^b^	0.662^b^	0.440^b^	0.149^b^	0.553^b^	0.469^b^		
Sex	0.283^b^	0.073	0.162^a^	0.032	0.074	0.174^b^	0.135^a^	0.101	
Age	− 0.085	0.007	0.004	0.006	0.089	− 0.005	0.044	0.03	− 0.123

*DER* difficulty in emotion regulation, *PA* physical abuse, *PN* physical neglect, *EA* emotional abuse, *EN* emotional neglect, *SA* sexual abuse, *NSSI* nonsuicidal self-injury

^a^*P* < 0.05

^b^*P* < 0.01

### Mediation analysis

**Fig. 1:**
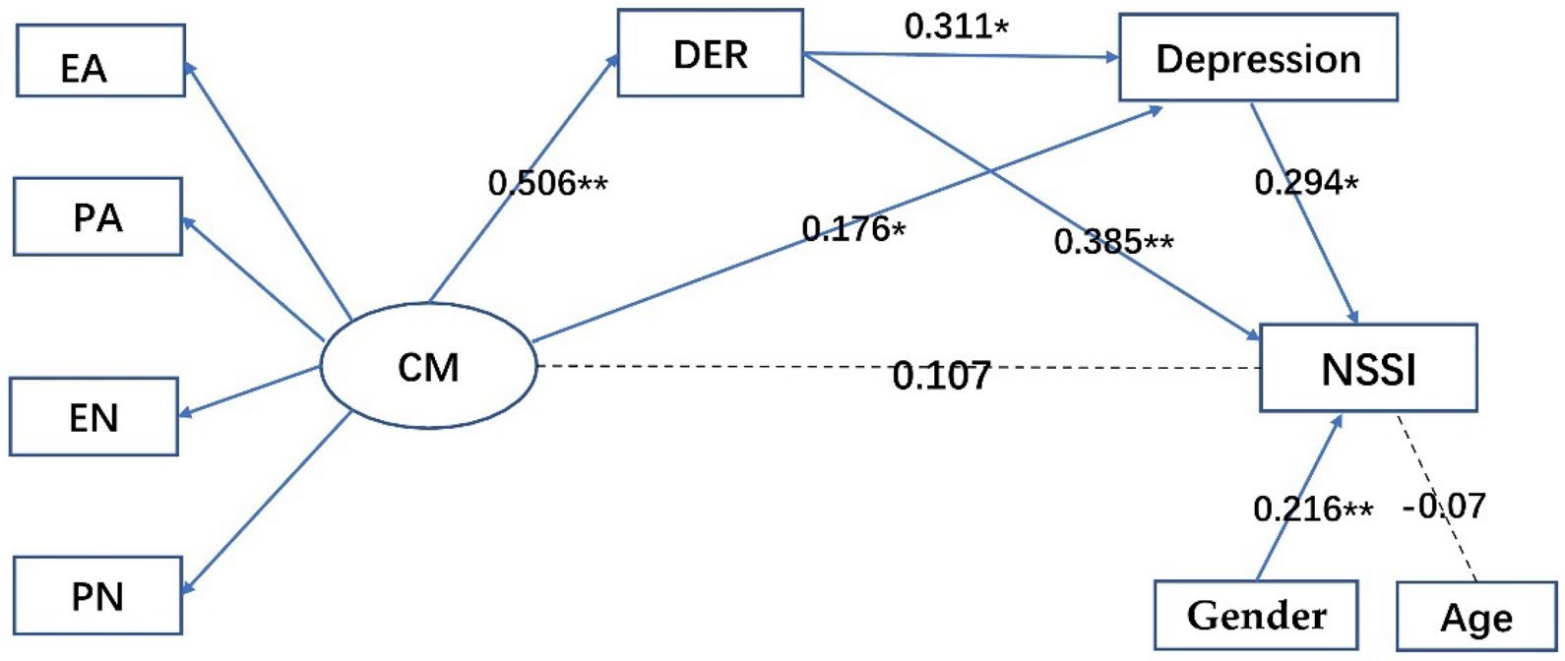
Results from path analysis on the hypothesized mediation model. Pathways between variables are indicated by standardized beta estimates. * *p* < 0.05, ** *p* < 0.01, *** *p* < 0.001. *DER* difficulty in emotion regulation, *PA* physical abuse, *PN* physical neglect, *EA* emotional abuse, *EN* emotional neglect, *SA* sexual abuse, *NSSI* nonsuicidal self-injury

As expected, the hypothesized chain mediation model was identified and fit the data well, with a *χ*^*2*^ (22, n = 224) = 47.564, *χ*^*2*^/*df* = 2.162*, p* < 0.001, a CFI = 0.95, a TLI = 0.919, and an RMSEA = 0.072. More specifically, the three indices showed a moderate fit. This model accounted for 27.9% (*P* < 0.001) of the total variance in NSSI frequency (Fig. 1 and Table 3). In line with our hypothesis, a mediating effect of DER and depression on the association between CM and NSSI was found. The total indirect effect was highly significant (*β* = 0.293; *P* = 0.010), while there was no significant direct effect in the pathway from CM to NSSI in the model (*β* = 0.107, *P* = 0.260). For the specific indirect effects, as predicted, DER and depression symptom severity were both found to be significant mediators. We also found a significant chain mediation effect for the DER to NSSI pathway via depression symptoms. Among the different indirect paths, DER was the strongest mediator, with an indirect effect of *β* = 0.195 (*P* = 0.010), and the indirect effect of depression symptom severity was *β* = 0.052 (*P* = 0.028). The indirect effect of DER on the association of CM and NSSI via depression symptom severity was *β* = 0.046 (*P* = 0.010). All total, direct, total indirect, and specific indirect effects are displayed in Table 3.

**Table 3:** Direct effects in the pathway from CM to NSSI and indirect effects through DER and depression

Path	Effect	*BC* 95% *CI*		*p*
		Lower	Upper	
Direct effect	0.107	− 0.067	0.315	0.260
Total indirect effect	0.293	0.149	0.488	0.010
Mediating effect of DER	0.195	0.091	0.376	0.010
Mediating effect of depression	0.052	0.009	0.101	0.028
Chain mediating effect	0.046	0.017	0.078	0.010
Total effect	0.400	0.184	0.678	0.010
R1	11.6%	0.044	0.265	0.010
R2	48.8%	0.310	0.801	0.010
R3	12.9%	0.015	0.427	0.029

R1: Chain mediating effect/Total effect; R2: Mediating effect of DER/Total effect; R3: Mediating effect of Depression/Total effect

## Discussion

This study aimed to examine the mediation pathway from CM exposure to adolescent NSSI behaviours based on a sample of adolescent patients in a psychiatric setting. The findings supported our hypothesis: CM exposure has indirect effects on the severity of NSSI via the mediating effect of DER severity and depressive symptoms. To our knowledge, this is the first investigation of the distal effects in the pathway from CM to NSSI severity via the chain mediating effects of DER and depression.

In our study, the prevalence of NSSI was over forty percent; girls had higher rates of NSSI, and depressive disorder was the primary psychiatric diagnosis. This is consistent with characteristics in previous adolescent samples [4, 35, 36]. Women were more likely to engage in NSSI than men in clinical samples [4], especially adolescent girls (16–19 years old) [37]. Further model comparison analysis found significant sex differences in the chain model. The chain mediation model explained girls well but not boys (Additional file 1: Table S2, Fig S2). Gender may play a role in NSSI mechanisms, and a recent review argues that CM seems less deleterious in males than females NSSI [7]. This difference may be due to lower levels of EA and EN (Additional file 1: Table S1), lower prevalence of NSSI (27.1%) to girls (51.1%), or smaller sample size (n = 48) for boys, which is lower than the recommended minimum sample size of 99. However, the main reason may be that only EA, except other CM types, was related to NSSI in our sample.

From our results, there were some unique features in the prevalence of different types of CM.The prevalence of SA in our sample was 12.5%, which was consistent with the result of a Chinese clinical sample study (12.5%) [38] and slightly higher than a meta-analysis for Chinese individuals (8.9% for women; 9.1% for men) [39]. In a review of SA studies worldwide, the prevalence in Asian countries was significantly lower than in other regions [40], and many reviewed studies were from China. Therefore, it might be evident that China did exhibit lower SA rates. It might explain why SA was not associated with NSSI severity in our study, unlike some studies in western countries [16]. The research regarding the relationship between SA and NSSI is somewhat inconclusive. Some studies have reported that sexual abuse was not directly associated with NSSI [9, 41]. Weierich and Nock, in contrast, found a relationship only between sexual abuse and NSSI and not between non-sexual abuse and NSSI [42]. The EA prevalence for girls in our study was significantly higher than that in North America (50.2% vs. 28.4%) and was twice that in an Asian sample (26.9%) [40]. However, the prevalence of EA for boys was comparable to the median prevalence rates in an Asian sample (29.2% vs. 33.2%) [40]. The prevalence of PA in our study was similar to recent international prevalence rates but lower than that in China a few years ago [43]. This is different from the prevalence in Asia, which is the same for boys and girls [40]. In conclusion, the girls in our study had higher rates of EA, lower rates of SA, and a similar prevalence of PA than those in the international sample and higher rates of EA and EN than the boys in our sample. These results may be related to China’s “one-child policy” and traditional Chinese parenting methods [44]. Chinese parents focus on their children's academic performance [45], not their emotional needs [46]. Compared with safety or sexual threats, children in Chinese families experience more emotional abuse and neglect, which may increase depression and emotional dysregulation [47].

At the same time, our study is different from that of Shenk, which may be due to different sample characteristics. Shenk’s study sample, recruited from a Child Protective Service (CPS) agency, had high levels of SA (58%), PA (34%) and posttraumatic stress symptoms [15], while our sample had lower rates of SA (12.5%), lower rates of Trauma and Stressor-Related Disorders (18.8%), and higher rates of EA (46.1%). Two recent studies from clinical and nonclinical samples were in good agreement with our results. A study in clinical samples found that DER mediated the relationship between EA and NSSI frequency and that EA was associated with NSSI but not SA. Another study in a nonclinical sample also found a partial mediating effect of EA, with a higher EA rate (72%) in participants with NSSI.

According to previous research results, CM is a distal associative factor of NSSI and a shared risk factor for depression and DER. For the properties of CM as a distal risk factor, the time bound for maltreatment was before the age of 12 years. Therefore, we speculate that CM mainly affects NSSI through these recent risk factors. The chain mediation model supports our hypothesis, and we found a mediation of the association between CM and NSSI with a significant indirect effect but no remaining direct effect. Whether CM is understood as a direct risk factor for NSSI is still debated [9, 48]. However, CM as a distal risk factor creates more indirect vulnerability for mental disorders, which increases the likelihood of engaging in NSSI [49].

DER was the strongest mediator, followed by depressive symptoms, in our model. Regardless, our findings are consistent with a previous study, which showed that the mediating effect of DER was higher than that of depression symptoms in the relationship between CM and NSSI [15, 50]. Early exposure to abusive or neglectful environments may disrupt children’s development of healthy emotion regulation skills and socioemotional competencies [51, 52]. Individuals with CM experience tend to use more maladaptive strategies, including inhibition and rumination [53], and have an impaired ability to use adaptive emotion regulation strategies, such as acceptance, reassessment, and problem-solving, which are associated with enhanced positive emotions and better mental health outcomes [53]. Without adaptive emotion regulation skills, self-harm behaviours may function as compensatory strategies to cope with overwhelming emotions. Specifically, self-harm may be used to distract oneself from distress and to regain a sense of control and self-efficacy [54]. We also proved a potential chain-mediated pathway of DER and depression in the relationship between CM and NSSI, which may add to our understanding of the relationship between CM and NSSI. The chain mediating effects of DER and depression was in line with the role of CM, as it impaired the development of self-regulation on emotional cognitive levels, resulting in poor emotional regulation and a depressogenic attributional style. Significantly, these cognitive and emotional sequelae of CM then increase the risk for the later development of depression symptoms [55, 56] and ultimately contribute to an increased possibility of NSSI [57]. Our findings potentially align with the biological conceptual model of NSSI [49]. According to this theory, early exposure to CM impairs the emotion regulation circuit of the brain through environmental-biological interactions in long-term adolescent development, causing DER and depression. Then, adolescents may adopt NSSI as a coping strategy for regulating aversive emotional experiences. Of course, this requires further imaging and biological research.

Our findings also have some clinical implications. First, our results contribute to the understanding that CM and NSSI are common among Chinese inpatient adolescents with psychiatric disorders, as shown in a recent study [35]. Many adolescents feel reluctant to talk about their CM or NSSI experiences in face-to-face settings. Hence, a mixed battery of self-reports and assessments by physicians may be more suitable in Chinese culture. Second, distal risk factors (i.e., CM) and proximal risk factors (especially DER) might work together to induce the onset of NSSI. Therefore, assessing CM, DER and depressive symptoms in adolescents with NSSI behaviours is also of practical importance, and we should enhance healthy emotion regulation strategies. Various components of DER, especially limited access to strategies [12], will be targeted for future NSSI interventions. Current interventions facilitating healthy emotion regulation might help modify maladaptive cognitions and behaviours [e.g., Dialectical Behaviour Therapy, DBT; Emotion Regulation Individual Therapy for Adolescents (ERITA)] [22, 58, 59]. However, more study is needed, as research on NSSI prevention is preliminary.

## Conclusions and limitations

Overall, this study explored the mechanism between the distal risk factors of CM and NSSI behaviour using a sample of inpatient adolescents in China, demonstrating a simple and chained mediating effect of DER and/or depressive symptoms. Importantly, our research shows that DER seems to be a mediator with a stronger indirect effect compared to depressive symptoms. Furthermore, we chose the CRSNSSI (DSM-5) as the NSSI evaluation index through structured interviews, which reflects the severity of NSSI better than self-rating NSSI scales. Despite such strengths, several limitations of the current study need to be acknowledged. First, the participants were patients in one hospital in Hangzhou, and male participants were underrepresented. Therefore, the sample source would limit the generalizability of these findings. Second, although we controlled the time boundaries of CM, we used cross-sectional data on DER and depression, and correlational data were used to test a causal model. Future research should utilize a longitudinal design while considering other possible mediators (e.g., life stressors). Third, we employed retrospective self-report questionnaires, and adolescents would unlikely be able to retrospectively report on maltreatment occurring in infancy, toddlerhood, or early childhood. Also, adolescents who are depressed, having difficulty regulating their emotions, and/or engaging in NSSI may be more likely to view their childhood experiences and family relationships more negatively. These might mean our data were not sufficiently objective. Therefore, these data may be affected by bias to some extent. Future studies should employ a large sample, multicentre, longitudinal design and adopt tools other than self-report questionnaires (e.g., expert opinions or other objective evidence). Future research should also examine subdomains of DER and their roles in NSSI behaviours. Because the recruitment period overlaps with the outbreak of COVID-19 in Hangzhou. Therefore, some impacts of COVID-19 need to be considered in our interpretation of results.

## Supplementary Information

**Additional file 1: Table S1.** Comparison of variables between boys and girls. **Table S2.** Direct effects in the pathway from CM to NSSI and indirect effects through DER and depression for girls. **Figure S1.** Path analysis on the hypothesized mediation model. **Figure S2.** Results from path analysis on the hypothesized mediation model for girls. Pathways between variables are indicated by standardized beta estimates. * *P* < 0.05, ** *P *< 0.01, *** *P* < 0.001. DER: difficulty in emotion regulation, PA: physical abuse, PN: physical neglect, EA: emotional abuse, EN: emotional neglect, SA: sexual abuse, NSSI: nonsuicidal self-injury. **Figure S3.** Results from path analysis on the hypothesized mediation model for boys. Pathways between variables are indicated by standardized beta estimates. * *P *< 0.05, **P < 0.01, *** *P *< 0.001. DER: difficulty in emotion regulation, PA: physical abuse, PN: physical neglect, EA: emotional abuse, EN: emotional neglect, SA: sexual abuse, NSSI: nonsuicidal self-injury.

## Data Availability

The data that support the findings of this study are available from the corresponding author upon reasonable request.

## References

[1] Nock MK (2010). Self-injury. Annu Rev Clin Psycho.

[2] Swannell SV, Martin GE, Page A, Hasking P, St John NJ (2014). Prevalence of nonsuicidal self-injury in nonclinical samples: systematic review meta-analysis and meta-regression. Suicide Life-Threat.

[3] Gillies D, Christou MA, Dixon AC, Featherston OJ, Rapti I, Garcia-Anguita A (2018). Prevalence and characteristics of self-harm in adolescents: meta-analyses of community-based studies 1990–2015. J Am Acad Child Psy.

[4] Bresin K, Schoenleber M (2015). Gender differences in the prevalence of nonsuicidal self-injury: a meta-analysis. Clin Psychol Rev.

[5] Meszaros G, Horvath LO, Balazs J (2017). Self-injury and externalizing pathology: a systematic literature review. Bmc Psychiatry.

[6] Mars B, Heron J, Klonsky ED, Moran P, O'Connor RC, Tilling K (2019). Predictors of future suicide attempt among adolescents with suicidal thoughts or non-suicidal self-harm: a population-based birth cohort study. Lancet Psychiatry.

[7] Liu RT, Scopelliti KM, Pittman SK, Zamora AS (2018). Childhood maltreatment and non-suicidal self-injury: a systematic review and meta-analysis. Lancet Psychiatry.

[8] Martin J, Raby KL, Labella MH, Roisman GI (2017). Childhood abuse and neglect, attachment states of mind, and non-suicidal self-injury. Attach Hum Dev.

[9] Brown RC, Heines S, Witt A, Braehler E, Fegert JM, Harsch D (2018). The impact of child maltreatment on non-suicidal self-injury: data from a representative sample of the general population. Bmc Psychiatry.

[10] Johnstone JM, Carter JD, Luty SE, Mulder RT, Frampton CM, Joyce PR (2016). Childhood predictors of lifetime suicide attempts and non-suicidal self-injury in depressed adults. Aust Nz J Psychiat.

[11] LeMoult J, Humphreys KL, Tracy A, Hoffmeister J, Ip E, Gotlib IH (2020). Meta-analysis: exposure to early life stress and risk for depression in childhood and adolescence. J Am Acad Child Psy.

[12] Wolff JC, Thompson E, Thomas SA, Nesi J, Bettis AH, Ransford B (2019). Emotion dysregulation and non-suicidal self-injury: a systematic review and meta-analysis. Eur Psychiat.

[13] Titelius EN, Cook E, Spas J, Orchowski L, Kivisto K, O'Brien K (2018). Emotion dysregulation mediates the relationship between child maltreatment and non-suicidal self-injury. J Aggress Maltreat T.

[14] Sim L, Adrian M, Zeman J, Cassano M, Friedrich WN (2009). Adolescent deliberate self-harm: linkages to emotion regulation and family emotional climate. J Res Adolesc.

[15] Shenk CE, Noll JG, Cassarly JA (2010). A multiple mediational test of the relationship between childhood maltreatment and non-suicidal self-injury. J Youth Adolesc.

[16] Serafini G, Canepa G, Adavastro G, Nebbia J, Murri MB, Erbuto D (2017). The relationship between childhood maltreatment and non-suicidal self-injury: a systematic review. Front Psychiatry.

[17] Hepp J, Carpenter RW, Störkel LM, Schmitz SE, Schmahl C, Niedtfeld I (2020). A systematic review of daily life studies on non-suicidal self-injury based on the four-function model. Clin Psychol Rev.

[18] Taylor PJ, Jomar K, Dhingra K, Forrester R, Shahmalak U, Dickson JM (2018). A meta-analysis of the prevalence of different functions of non-suicidal self-injury. J Affect Disorders.

[19] Mm L (1993). Cognitive-behavioral treatment of borderline personality disorder.

[20] Crowell SE, Beauchaine TP, Linehan MM (2009). A biosocial developmental model of borderline personality: elaborating and extending linehan’s theory. Psychol Bull.

[21] Gratz KL, Roemer L (2004). Multidimensional assessment of emotion regulation and dysregulation: development, factor structure, and initial validation of the difficulties in emotion regulation scale. J Psychopathol Behav.

[22] Bjureberg J, Sahlin H, Hedman-Lagerlof E, Gratz KL, Tull MT, Jokinen J (2018). Extending research on emotion regulation individual therapy for adolescents (ERITA) with nonsuicidal self-injury disorder: open pilot trial and mediation analysis of a novel online version. Bmc Psychiatry.

[23] Folk JB, Zeman JL, Poon JA, Dallaire DH (2014). A longitudinal examination of emotion regulation: pathways to anxiety and depressive symptoms in urban minority youth. Child Adol Ment H-Uk.

[24] Crow T, Cross D, Powers A, Bradley B (2014). Emotion dysregulation as a mediator between childhood emotional abuse and current depression in a low-income African-American sample. Child Abuse Neglect.

[25] Ledermann T, Kenny DA (2012). The common fate model for dyadic data: variations of a theoretically important but underutilized model. J Fam Psychol.

[26] APA (2013). Diagnostic and statistical manual of mental disorders (DSM-5).

[27] Bernstein DP, Stein JA, Newcomb MD, Walker E, Pogge D, Ahluvalia T (2003). Development and validation of a brief screening version of the childhood trauma questionnaire. Child Abuse Neglect.

[28] Li S, Zhao F, Yu G (2020). Childhood emotional abuse and depression among adolescents: roles of deviant peer affiliation and gender. J Interpers Violence.

[29] Jugessur R, Zhang Y, Qin X, Wang M, Lu X, Sun J (2021). Childhood maltreatment predicts specific types of dysfunctional attitudes in participants with and without depression. Front Psychiatry.

[30] Li J, Han ZR, Gao MM, Sun X, Ahemaitijiang N (2018). Psychometric properties of the Chinese version of the difficulties in emotion regulation scale (DERS): factor structure, reliability, and validity. Psychol Assess.

[31] Kroenke K, Spitzer RL, Williams JB (2001). The PHQ-9: validity of a brief depression severity measure. J Gen Intern Med.

[32] Zuckerbrot RA, Amy C, Jensen PS, Stein REK, Laraque D (2018). Guidelines for adolescent depression in primary care (GLAD-PC): part I. practice preparation, identification, assessment, and initial management. Pediatrics.

[33] Leung D, Mak YW, Leung SF, Chiang V, Loke AY (2020). Measurement invariances of the PHQ-9 across gender and age groups in Chinese adolescents. Asia-Pac Psychiat.

[34] Browne MW, Cudeck R (1992). Alternative ways of assessing model fit. Sociol Method Res.

[35] Wang L, Liu J, Yang Y, Zou H (2021). Prevalence and risk factors for non-suicidal self-injury among patients with depression or bipolar disorder in China. Bmc Psychiatry.

[36] Tuisku V, Pelkonen M, Kiviruusu O, Karlsson L, Ruuttu T, Marttunen M (2009). Factors associated with deliberate self-harm behaviour among depressed adolescent outpatients. J Adolesc.

[37] Wilkinson PO, Qiu T, Jesmont C, Neufeld S, Kaur SP, Jones PB (2022). Age and gender effects on non-suicidal self-injury, and their interplay with psychological distress. J Affect Disorders.

[38] Zhang TH, Chow A, Wang LL, Yu JH, Dai YF, Lu X (2013). Childhood maltreatment profile in a clinical population in China: a further analysis with existing data of an epidemiologic survey. Compr Psychiat.

[39] Ma Y (2018). Prevalence of childhood sexual abuse in China: a meta-analysis. J Child Sex Abus.

[40] Moody G, Cannings-John R, Hood K, Kemp A, Robling M (2018). Establishing the international prevalence of self-reported child maltreatment: a systematic review by maltreatment type and gender. BMC Public Health.

[41] David KE, Anne M (2008). Childhood sexual abuse and non-suicidal self-injury: meta-analysis. Br J Psychiatry.

[42] Weierich MR, Nock MK (2008). Posttraumatic stress symptoms mediate the relation between childhood sexual abuse and nonsuicidal self-injury. J Consult Clin Psych.

[43] Ji K, Finkelhor D (2015). A meta-analysis of child physical abuse prevalence in China. Child Abuse Neglect.

[44] Kim SY, Wang Y, Orozco-Lapray D, Shen Y, Murtuza M (2013). Does, "tiger parenting" exist? parenting profiles of Chinese Americans and adolescent developmental outcomes. Asian Am J Psychol.

[45] Sin-Sze CC, PE M (2011). Parents' involvement in children's learning in the United States and China: implications for children’s academic and emotional adjustment. Child Dev.

[46] Cui L, Zhang X, Han ZR (2021). perceived child difficultness, emotion dysregulation, and emotion-related parenting among chinese parents. Fam Process.

[47] Gruhn MA, Compas BE (2020). Effects of maltreatment on coping and emotion regulation in childhood and adolescence: a meta-analytic review. Child Abuse Neglect.

[48] Paul E, Ortin A (2019). Psychopathological mechanisms of early neglect and abuse on suicidal ideation and self-harm in middle childhood. Eur Child Adoles Psy.

[49] Kaess M, Hooley JM, Klimes-Dougan B, Koenig J, Plener PL, Reichl C (2021). Advancing a temporal framework for understanding the biology of nonsuicidal self- injury: an expert review. Neurosci Biobehav Rev.

[50] Peh CX, Shahwan S, Fauziana R, Mahesh MV, Sambasivam R, Zhang Y (2017). Emotion dysregulation as a mechanism linking child maltreatment exposure and self-harm behaviors in adolescents. Child Abuse Neglect.

[51] Maughan A, Cicchetti D (2002). Impact of child maltreatment and interadult violence on childrens emotion regulation abilities and socioemotional adjustment. Child Dev.

[52] Yates TM (2009). Developmental pathways from child maltreatment to nonsuicidal self-injury.

[53] Nock MK (2009). Understanding nonsuicidal self-injury: origins, assessment, and treatment.

[54] Weissman DG, Bitran D, Miller AB, Schaefer JD, Sheridan MA, McLaughlin KA (2019). Difficulties with emotion regulation as a transdiagnostic mechanism linking child maltreatment with the emergence of psychopathology. Dev Psychopathol.

[55] Lang CM, Sharma-Patel K (2011). The relation between childhood maltreatment and self-injury: a review of the literature on conceptualization and intervention. Trauma Violence Abus.

[56] Huh HJ, Kim KH, Lee H, Chae J (2017). The relationship between childhood trauma and the severity of adulthood depression and anxiety symptoms in a clinical sample: the mediating role of cognitive emotion regulation strategies. J Affect Disorders.

[57] Schierholz A, Krüger A, Barenbrügge J, Ehring T (2016). What mediates the link between childhood maltreatment and depression? the role of emotion dysregulation, attachment, and attributional style. Eur J Psychotraumato.

[58] Baiden P, Stewart SL, Fallon B (2017). The mediating effect of depressive symptoms on the relationship between bullying victimization and non-suicidal self-injury among adolescents: findings from community and inpatient mental health settings in ontario. Canada, Psychiat Res.

[59] Tebbett-Mock AA, Saito E, McGee M, Woloszyn P, Venuti M (2020). Efficacy of dialectical behavior therapy versus treatment as usual for acute-care inpatient adolescents. J Am Acad Child Adolesc Psychiatry.

[60] Adrian M, McCauley E, Berk MS, Asarnow JR, Korslund K, Avina C (2019). Predictors and moderators of recurring self-harm in adolescents participating in a comparative treatment trial of psychological interventions. J Child Psychol Psyc.

