# Supplementary material for: Child maltreatment exposure and adolescent nonsuicidal self-injury: the mediating roles of difficulty in emotion regulation and depressive symptoms
Source: Child Adolesc Psychiatry Ment Health. 2023 Jan 28;17:16. doi: 10.1186/s13034-023-00557-3 (PMC9883603; doi:10.1186/s13034-023-00557-3)
Supplement: Supplementary file 1 — Additional file 1: Table S1. Comparison of variables between boys and girls. Table S2. Direct effects in the pathway from CM to NSSI and indirect effects through DER and depression for girls. Figure S1. Path analysis on the hypothesized mediation model. Figure S2. Results from path analysis on the hypothesized mediation model for girls. Pathways between variables are indicated by standardized beta estimates. * P < 0.05, ** P < 0.01, *** P < 0.001. DER: difficulty in emotion regulation, PA: physical abuse, PN: physical neglect, EA: emotional abuse, EN: emotional neglect, SA: sexual abuse, NSSI: nonsuicidal self-injury. Figure S3. Results from path analysis on the hypothesized mediation model for boys. Pathways between variables are indicated by standardized beta estimates. * P < 0.05, **P < 0.01, *** P < 0.001. DER: difficulty in emotion regulation, PA: physical abuse, PN: physical neglect, EA: emotional abuse, EN: emotional neglect, SA: sexual abuse, NSSI: nonsuicidal self-injury. [file 13034_2023_557_MOESM1_ESM.docx]

**Additional file**

Comparison of the boy and girl samples for the chain mediation model

A sex difference comparison of the participants' variables is presented in Table 1. The prevalence of NSSI in girls was significantly higher than that in boys (*χ^2^*=8.38*, P*=0.004). The boys scored lower than the girls on the EA subscale (*t* = -2.45, *P*=0.015), the EN subscale (*t* = -2.64, *P*=0.009), and the PN subscale (*t*= -2.03, *P*=0.043) but not the DERS (*P*> 0.05) and PHQ-9 (*P*> 0.05).

To further examine differences between the boy and girl samples, tests of invariance were conducted according to tests described by Byrne [1]. To test for configural invariance and whether different sexes were similar across samples, the same model was applied to the combined data for boys and girls. An acceptable model fit was found for the combined configural model: *χ^2^* (52, n=224) =78.1, χ^2^/df =1.50, NFI=0.863, CFI=0.948, and RMSEA= 0.048. The chi-square difference test showed the following: Δχ^2^ (13) =31.76, *P*=0.03. As another alternative to the chi-square difference test, Byrne [1]suggests comparing CFI values. The difference in the CFI statistics (0.948 vs. 0.910) exceeded the value of 0.01 set as evidence for invariance by Cheung and Rensvold [2]. Thus, based on the chi-square difference test and the test of differences in the CFI, we concluded that the sex factor was not invariant in our samples. The hypothesized chain mediation model was identified and provided a good fit for the girl sample (*χ^2^* (16, n=176) =29.366, *P* =0.022, CFI=0.962, TLI=0.933, and RMSEA=0.069), which was consistent with that for the entire sample (Table 2 and Figure 2). However, the boy sample had a poor model fit (*χ^2^* (16, n=48) =20.201, *P*=0.211, CFI=0.97, TLI=0.947, and RMSEA=0.075), and the hypothesized chain mediation model wasn’t identified (Figure 3).

**Table S1.** Comparison of variables between boys and girls.

| **Variable** | boys (n=48) | girls (n=176) | *t/χ^2^* | *P* |
| --- | --- | --- | --- | --- |
| **Age (years)**, *Mean (SD)* | 15.73(1.36) | 15.18(1.92) | 1.85 | 0.066 |
| **DERS**, *Mean (SD)* | 116.15(20.73) | 120.16(23.18) | -1.09 | 0.278 |
| **Childhood Maltreatment** |  |  |  |  |
| EA, *Mean (SD)* | 11.19(5.6) | 13.24(5.02) | -2.45 | 0.015 |
| PA, *Mean (SD)* | 7.65(3.74) | 7.93(3.61) | -0.47 | 0.636 |
| SA, *Mean (SD)* | 5.73(2.08) | 6.22(2.83) | -1.11 | 0.267 |
| EN, *Mean (SD)* | 12.77(5.55) | 15.1(5.38) | -2.64 | 0.009 |
| PN, *Mean (SD)* | 8.54(4.2) | 9.91(4.14) | -2.03 | 0.043 |
| EA, *n (%)* | 14(29.2%) | 89(50.6%) | 6.96 | 0.008 |
| PA, *n (%)* | 11(22.9%) | 41(23.3%) | 0.003 | 0.956 |
| SA, *n (%)* | 4(8.3%) | 24(13.6%) | 0.97 | 0.325 |
| EN, *n (%)* | 14(29.2%) | 94(53.4%) | 8.87 | 0.003 |
| PN, *n (%)* | 14(29.2%) | 83(47.2%) | 4.97 | 0.026 |
| **Depression (**PHQ-9), Mean (SD) | 17.53(5.46) | 18.55(5.44) | -1.16 | 0.249 |
| **NSSI (CRSNSSI)****/n (%)* | 13(27.1%) | 90(51.1%) | 8.38 | 0.004 |

DERS: Difficulties in Emotion Regulation Scale, PA: physical abuse, PN: physical neglect, EA: emotional abuse, EN: emotional neglect, SA: sexual abuse, CRSNSSI: Clinician-Rated Severity of NonSuicidal Self-Injury, *: The number that meets the DSM-5 diagnostic criteria for NSSI

**Table S2.** Direct effects in the pathway from CM to NSSI and indirect effects through DER and depression for girls.

| Path | Effect | *BC* 95% *CI* | | *p* |
| --- | --- | --- | --- | --- |
|  |  | Lower | Upper |  |
| Direct effect | 0.091 | -0.106 | 0.303 | 0.352 |
| Total indirect effect | 0.273 | 0.145 | 0.468 | <0.001 |
| Mediating effect of DER | 0.178 | 0.078 | 0.360 | <0.001 |
| Mediating effect of depression | 0.050 | 0.022 | 0.110 | <0.001 |
| Chain mediating effect | 0.044 | 0.006 | 0.099 | 0.029 |
| Total effect | 0.363 | 0.146 | 0.664 | 0.001 |
| R1 | 13.7% | 0.049 | 0.403 | 0.002 |
| R2 | 49.1% | 0.249 | 0.953 | 0.003 |
| R3 | 12.2% | 0.007 | 0.532 | 0.040 |

R1: Chain mediating effect/Total effect; R2: Mediating effect of DER/Total effect; R3: Mediating effect of Depression/Total effect


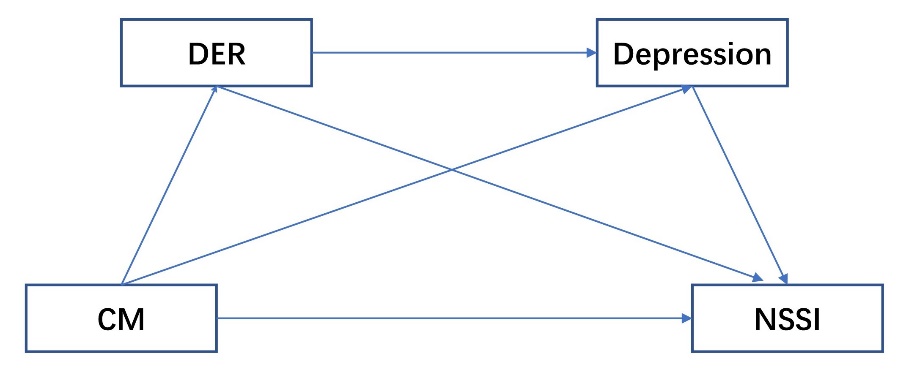


**Figure S1.** Path analysis on the hypothesized mediation model.


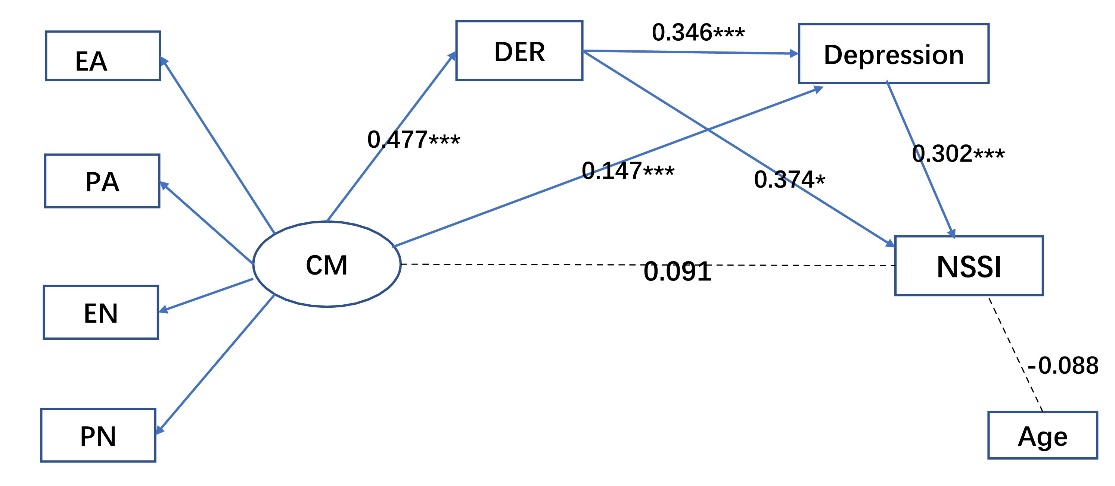


**Figure S2.** Results from path analysis on the hypothesized mediation model for girls. Pathways between variables are indicated by standardized beta estimates. * *P* < 0.05, ** *P* < 0.01, *** *P* < 0.001. DER: difficulty in emotion regulation, PA: physical abuse, PN: physical neglect, EA: emotional abuse, EN: emotional neglect, SA: sexual abuse, NSSI: nonsuicidal self-injury.


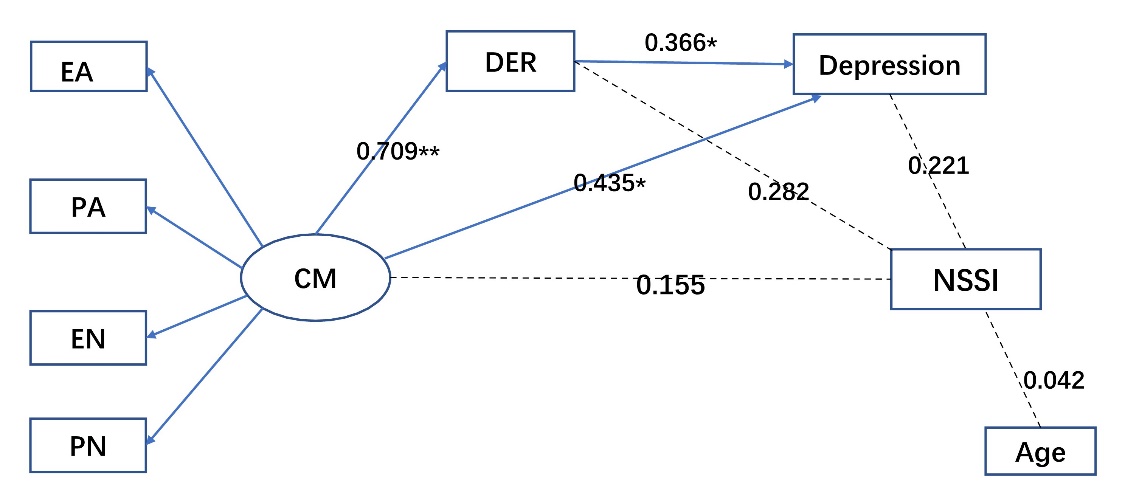


**Figure S3.** Results from path analysis on the hypothesized mediation model for boys. Pathways between variables are indicated by standardized beta estimates. * *P* < 0.05, ***P* < 0.01, *** *P* < 0.001. DER: difficulty in emotion regulation, PA: physical abuse, PN: physical neglect, EA: emotional abuse, EN: emotional neglect, SA: sexual abuse, NSSI: nonsuicidal self-injury.

**References**

1. Byrne BM. Structural equation modeling with AMOS. New York, NY: Routledge, 2010.

2. Cheung GW, Rensvold RB. Evaluating Goodness-of-Fit Indexes for Testing Measurement Invariance, Structural equation modeling 2002;9:233-255.
